# The Association between Aortic Valve Stenosis and a Subsequent Diagnosis of Depression in Germany

**DOI:** 10.3390/jcm13185525

**Published:** 2024-09-18

**Authors:** Sven Thomas Niepmann, Christoph Roderburg, Mark Luedde, Georg Nickenig, Sven H. Loosen, Karel Kostev

**Affiliations:** 1 Heart Center Bonn, Clinic for Internal Medicine II, University Hospital Bonn, 53127 Bonn, Germany; 2 Department of Gastroenterology, Hepatology and Infectious Diseases, Medical Faculty of Heinrich Heine University Duesseldorf, University Hospital Duesseldorf, 40225 Duesseldorf, Germany; 3 Department of Cardiology, Christian Albrechts University of Kiel, 24118 Kiel, Germany; 4 Epidemiology, IQVIA, 60549 Frankfurt, Germany

**Keywords:** aortic valve stenosis, depression, epidemiology, cardiovascular disease, mood disorders

## Abstract

**Background/Objectives**: Aortic valve stenosis (AS) represents one of the most common valve diseases in the western world. It often leads to severe symptoms that can lead to a restriction of everyday life and thus to psychological stress. Therefore, we aimed to investigate the association between AS and depression in outpatients in Germany. **Methods**: The IQVIA^TM^ Disease Analyzer database was used to identify 14,681 individuals with non-rheumatic AS (ICD-10: I35.0 or I35.2). They were propensity score matched (1:1) based on age, sex, average yearly consultation frequency during the follow-up, and co-diagnoses to 14,681 patients without AS. Cox regression models were used to analyze the association between aortic stenosis and depression. **Results**: Within the follow-up period of up to 10 years, depression was diagnosed in 20.6% of AS patients compared to 20.0% in the matched cohort (*p* = 0.351). In the regression analysis, we were not able to discover an association between AS and a subsequent diagnosis of depression (HR: 1.03; 95% CI: 0.96–1.11). This effect was consistent among different age and sex groups. **Conclusions**: In the broad population of patients treated outside of hospital settings in Germany, AS was not associated with a higher incidence of depression.

## 1. Introduction

Aortic valve stenosis (AS) is the most prevalent type of valve disease requiring therapeutical intervention in western countries [1,2]. In younger patients, AS can develop as a consequence of congenital bicuspid aortic valve disease [3]. Cases of rheumatic AS, in association with rheumatic mitral stenosis, are rare in western countries since antibiotic treatment upon Streptococcus infections is available. In underdeveloped countries, rheumatic AS remains prevalent [4]. The most common cause of AS in western countries is age-related progressive fibrosis and calcification of the aortic valve [5]. Due to an increasingly older society, the incidence is expected to multiply further within the next years [2,6].

The first signs of AS are often fibrosis of the aortic valve in echocardiographic images. Of these patients, approximately 15% develop AS with a narrowing of the aortic valve orifice area. Once mild or moderate stenosis is diagnosed, nearly every patient develops severe AS. Between the first diagnosis and that of severe AS, many years may pass [7]. To date, medical treatments that are able to slow down or prevent the further progression of AS have not found their way into clinical practice. Therefore, current guidelines suggest regular follow-ups and the strict monitoring of patients with moderate AS [8]. Once the orifice area is critically narrowed (<1 cm^2^), severe AS leads to left ventricular outflow obstruction, which causes inadequate cardiac output [7]. Patients with AS are severely restricted in their daily life: symptoms such as dyspnea, angina pectoris, dizziness, syncope, and reduced exercise capacity are typical of AS. An increased afterload promotes concentric hypertrophy, followed by dilatation of the left ventricle and heart failure in the advanced stages of severe AS [9]. Heart failure leads to frequent hospitalizations, and the mortality of symptomatic AS rises up to 50% over two years [10]. Therefore, valve replacement, either via open surgery or trans-catheter valve replacement, is required in patients with severe AS [8].

Because of the significant restriction in daily life that patients with AS suffer from, we hypothesized that AS could be associated with increased incidence of depression. We therefore used data from primary care practices to analyze a potential association between AS and a subsequent diagnosis of depression.

## 2. Materials and Methods

### 2.1. Database

Data from the Disease Analyzer database (IQVIA) were used to create this retrospective cohort study. This database has been extensively used in several previous studies, including those focusing on heart diseases [11,12] and depression [13,14]. These methods have therefore been described in detail. Briefly, the database contains de-identified information on diagnoses and prescriptions, as well as the fundamental medical and demographic data from computer systems used in office-based practices [15]. The database covers approximately 3–5% of all outpatient practices in Germany. The sampling method for the Disease Analyzer database uses statistics from the German Medical Association to determine the panel design according to the specialist group, the German federal state, the community size category, and the age of the physician. It has previously been shown that the panel of practices included in the Disease Analyzer database is representative of general and specialized practices in Germany [15].

### 2.2. Ethical Approval

The “Disease Analyzer” database used for analysis contains only anonymized data from electronic patient records. Patient data were analyzed in aggregated form; individual data were not available. An individual consent form was not obtained, following national and European legislation.

### 2.3. Study Population

**Figure 1:**
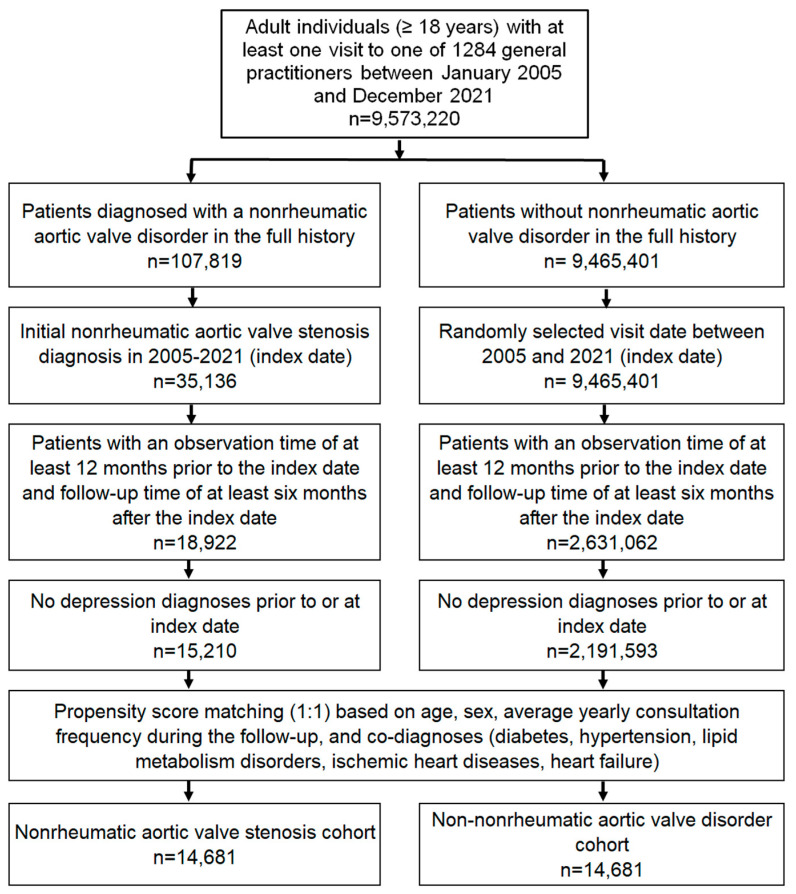
Selection of study patients.

This study included patients aged ≥18 years with a diagnosis of non-rheumatic aortic valve stenosis (AS, ICD-10: I35.0, I35.2) in 1284 general practices in Germany between January 2005 and December 2021 (index date; see Figure 1). The first documented diagnosis of AS in this time period was considered as the index date. Further inclusion criteria included an observation time of at least 12 months prior to the index date and a follow-up time of at least six months after the index date. Patients with a diagnosis of depression prior to or at the index date were excluded.

After applying similar inclusion criteria, individuals without AS were matched to AS patients using propensity score matching (1:1) based on age, sex, average yearly consultation frequency during follow-up and co-diagnoses (diabetes (ICD-10: E10-E14; hypertension (ICD-10: I10), lipid metabolism disorders (ICD-10: E78), ischemic heart diseases (ICD-10: I20-I25), and heart failure (ICD-10: I50)). For the non-AS cohort, the index date was a randomly selected visit between January 2005 and December 2021 (Figure 1).

### 2.4. Study Outcomes and Statistical Analyses

Statistical analyses were performed as previously described [14]. The main outcome of the study was the initial diagnoses of depression (ICD-10: F32, F33) up to ten years following the index date as a function of AS. Differences in the sample characteristics and diagnosis prevalence between the AS and non-AS cohorts were compared using the Wilcoxon signed rank test for continuous variables, the McNemar test for categorical variables with two categories, and the Stuart–Maxwell test for categorical variables with more than two categories. The 10-year cumulative incidence of depression in the cohorts with and without AS was further studied via Kaplan–Meier curves, and these curves were compared using the log-rank test. Finally, an univariable Cox regression analysis was conducted to assess the association between AS and depression. The results of the Cox-regression model are displayed as hazard ratios (HRs) and 95% confidence intervals (CIs). Additionally, Cox regression analyses were conducted separately for men and women, as well as for five age groups. A *p*-value of <0.05 was considered to be statistically significant. Analyses were carried out using SAS version 9.4 (SAS Institute, Cary, NC, USA).

## 3. Results

### 3.1. Basic Characteristics of the Study Sample

The here-introduced study included 14,681 individuals with AS and 14,681 persons without AS. The basic characteristics of the study cohort are displayed in Table 1. The mean age was 75 years (standard deviation (SD): 11), and 44.2% of patients were female. On average, 10 visits to their general practitioners’ offices have been recorded per year during the follow-up. Because of the matched pairs design, no significant differences could be observed between the two groups in terms of age, sex, and visit frequency. Co-morbidities such as cardiovascular risk factors and heart failure were equally distributed between the two groups (Table 1).

### 3.2. Association between AS and a Subsequent Diagnosis of Depression

**Figure 2:**
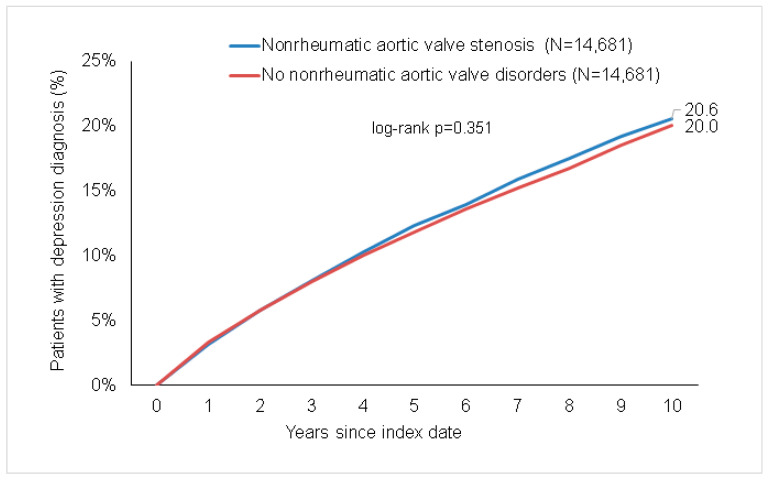
Cumulative incidence of depression in individuals with and without aortic valve stenosis.

After up to 10 years of follow-up, depression was diagnosed in 20.6% of AS patients. In the matched non-AS cohort, 20.0% of patients were diagnosed with depression. A difference between the two groups could not be observed (*p* = 0.351; Figure 2). In the regression analysis, there were no significant associations between AS and a subsequent depression diagnosis (HR: 1.03; 95% CI: 0.96–1.11; Table 2). Furthermore, no significant associations between both conditions were observed in the age- and sex-stratified analyses (Table 2).

## 4. Discussion

The slowly progressing narrowing of the aortic valve’s orifice area leads to a significant burden on the patient’s morbidity and quality of life [16]. However, in the present study, the incidence of depression was not significantly increased in patients with AS compared to individuals without a diagnosis of AS. Interestingly, this finding was also consistent in the prespecified sex and age groups. A non-significant trend towards a higher incidence of depression could be observed in younger patients aged under 50 with a diagnosis of AS compared to patients without AS. In line with the literature, both groups showed an increased incidence of depression in women compared to men [17].

In recent years, depression has emerged as an important risk factor for cardiovascular disease. Especially in heart failure, diagnosis of concomitant depression is frequent, and the incidence is even higher than in patients with different types of cancer [13]. In heart failure, depression and anxiety are underdiagnosed and associated with increased mortality [18]. Various studies have also investigated the connection between depression and AS. However, most of these studies have focused on patients undergoing aortic valve replacement. These patients suffer from symptomatic severe aortic valve stenosis. It has been shown that depression in these patients is common and that depression is associated with an increased mortality after valve replacement [19,20]. In contrast, in our collective, we examined all patients with a diagnosis of aortic valve stenosis regardless of the disease stage. Likewise, in contrast to the existing studies, we compared patients with AS with patients without AS. Studies have also shown a rapid improvement in anxiety and depression in elderly patients after transcatheter aortic valve replacement [21]. As expected, this improvement is time-delayed in patients with advanced AS stages who already show extra-valvular cardiac damage [22]. Interestingly, the risk of developing a new depression after trans-catheter valve replacement is also reduced in the first months after intervention compared to patients after surgical valve replacement [23]. It could therefore be hypothesized that the improved treatment methods have an influence on the development of patients’ mental health. After the introduction of TAVR, twice as many patients with severe AS were treated compared to the last decade [24] since it is a low-risk procedure which is also suitable for older patients or patients with severe comorbidities [10]. TAVR leads to the rapid improvement of symptoms and of quality of life [25]. This could also support the results of our study. It can be assumed that patients will receive valve replacement more quickly and that patients who are unsuitable for surgical aortic valve replacement will also be treated. As a result, patients do not develop longer histories of illness; therefore, the rate of patients with depression and AS is not increased compared to patients without AS. On the other hand, it could be concluded from our data that depression could be underdiagnosed in patients with AS. This has already been demonstrated by Druti et al. in 2018. They reported that in a collective of over 1000 patients with severe AS, with a mean age of 81 years, about 31% had a positive screening test for depression, whereas in only 8.6% depressions were already documented in their medical record [19].

It is also important to note that the incidence of depression in our control group is also 20% (Figure 2). This could be due to the fact that similar to patients with AS, the patients of the control group also suffer from significant comorbidities that can also lead to depression. For example, diabetes mellitus [26], heart failure [27], and high blood pressure [28] have been shown to increase the incidence of depression. Thus, it could be hypothesized that the incidence of depression might be increased in patients with aortic valve stenosis compared to healthy individuals without the mentioned comorbidities and that our results might therefore represent a false negative. On the one hand, these comorbidities are important risk factors of aortic valve stenosis; thus, a coincidence is very common [29,30,31]; a comparison to healthy individuals does not seem reasonable. On the other hand, these facts should cause us to pay attention to the simultaneous presence of depressive symptoms in patients with aortic valve stenosis and the accompanying risk factors.

We would like to point out some imitations of our study. First, the Disease Analyzer database records only ICD-10 codes. This may carry the risk of the incorrect coding or non-coding of various diagnoses. Second, there are no echocardiographic data and, therefore, no staging of AS. Patients with mild or moderate stenosis are often asymptomatic and are therefore not predestined to develop depressive symptoms. Third, the database has no information on surgical or interventional treatment of AS. It could be that some of the patients with AS have already undergone valve replacement, and this might have led to an improvement in their depressive symptoms. Fourth, the database does not contain data on lifestyle factors such as smoking behavior, alcohol intake, family status, etc., which could influence mood disorders. Fifth, unfortunately, it is not possible to display time sequences with this database. Therefore, we cannot clarify whether the patients with depression are those who have a long history of aortic valve stenosis. Finally, the disease database analyzer does not include data from hospitals. Data from cardiological practices, where most of the patients with AS are treated, are available in the database but could not be used for this study as cardiologists only rarely document depression.

## 5. Conclusions

In summary, no association between AS and depression was found in this study. However, on one hand, depression is underdiagnosed in AS, and depression leads to an increased mortality after aortic valve replacement; on the other hand, frequent risk factors and comorbidities of AS have been associated with depression. Therefore, targeted screening for mood disorders in patients with AS might nevertheless be reasonable.

## Figures and Tables

**Table 1:** Baseline characteristics of the study sample (after propensity score matching).

Variable	Proportion among Individuals with Aortic Valve Stenosis (%) *n* = 14,681	Proportion among Individuals without Aortic Valve Disorders (%) *n* = 14,681	*p*-Value
Age (mean, SD)	74.7 (11.1)	74.6 (11.2)	0.459
Age ≤ 50	3.4	3.4	
Age 51–60	7.3	7.5	
Age 61–70	17.6	17.9	0.875
Age 71–80	38.9	38.9	
Age > 80	32.8	32.3	
Women	44.2	44.3	0.879
Men	55.8	55.7	
Number of physician visits per year during the follow-up (mean, SD)	9.7 (3.5)	9.7 (3.5)	0.918
Diagnoses documented within 12 months prior to or at index date			
Diabetes	36.6	36.7	0.856
Hypertension	78.8	78.9	0.830
Lipid metabolism disorders	53.2	53.3	0.879
Ischemic heart diseases	10.1	10.1	0.985
Heart failure	22.3	22.3	1.000

Proportions of patients given in % unless otherwise indicated. SD: standard deviation.

**Table 2:** Association between aortic valve stenosis and subsequent depression in patients followed up in general practices in Germany (univariable Cox regression models).

	Incidence (Cases per 1000 Patients Years) in Individuals with Aortic Valve Stenosis (%)	Incidence (Cases per 1000 Patients Years) in Individuals without Aortic Valve Disorders (%)	HR (95% CI)	*p*-Value
Total	25.4	25.0	1.03 (0.96–1.11)	0.351
Age ≤ 50	34.4	29.2	1.23 (0.91–1.67)	0.185
Age 51–60	27.6	27.2	1.03 (0.83–1.29)	0.777
Age 61–70	17.6	17.6	1.01 (0.85–1.21)	0.904
Age 71–80	24.4	24.3	1.02 (0.91–1.14)	0.726
Age > 80	32.1	31.6	1.03 (0.90–1.17)	0.701
Women	31.7	31.3	1.03 (0.94–1.14)	0.512
Men	20.7	20.3	1.04 (0.94–1.15)	0.487

## Data Availability

The datasets used and/or analyzed during the current study are available from the corresponding author upon reasonable request.
